# Thyroid Autoimmunity is Associated with Decreased Cytotoxicity T Cells in Women with Repeated Implantation Failure

**DOI:** 10.3390/ijerph120910352

**Published:** 2015-08-25

**Authors:** Chunyu Huang, Peiyan Liang, Lianghui Diao, Cuicui Liu, Xian Chen, Guangui Li, Cong Chen, Yong Zeng

**Affiliations:** 1 Fertility Center, Shenzhen Zhongshan Urology Hospital, Shenzhen 518045, China; E-Mails: huangchunyu200502@aliyun.com (C.H.); victoria_lpy@hotmail.com (P.L.); diaolianghui@gmail.com (L.D.); lucyliucuicui@126.com (C.L.); x_chen2011@163.com (X.C); liguangui@hotmail.com (G.L.); congchen2015@sina.com (C.C.); 2 Shenzhen Key Laboratory of Reproductive Immunology for Peri-implantation, Shenzhen 518045, China; 3 Shenzhen Zhongshan Institute for Reproductive Medicine and Genetics, Shenzhen 518045, China

**Keywords:** infertility, repeated implantation failure, thyroid autoimmunity, lymphocytes

## Abstract

Thyroid autoimmunity (TAI), which is defined as the presence of autoantibodies against thyroid peroxidase (TPO) and/or thyroglobulin (TG), is related to repeated implantation failure (RIF). It is reported that TAI was involved in reproductive failure not only through leading thyroid function abnormality, but it can also be accompanied with immune imbalance. Therefore, this study was designed to investigate the association of thyroid function, immune status and TAI in women with RIF. Blood samples were drawn from 72 women with RIF to evaluate the prevalence of TAI, the thyroid function, the absolute numbers and percentages of lymphocytes. The prevalence of thyroid function abnormality in RIF women with TAI was not significantly different from that in RIF women without TAI (χ^2^ = 0.484, *p* > 0.05). The absolute number and percentage of T cells, T helper (Th) cells, B cells and natural killer (NK) cells were not significantly different in RIF women with TAI compared to those without TAI (all *p* > 0.05). The percentage of T cytotoxicity (Tc) cells was significantly decreased in RIF women with TAI compared to those without TAI (*p* < 0.05). Meanwhile, Th/Tc ratio was significantly increased (*p* < 0.05). These results indicated that the decreased Tc percentage and increased Th/Tc ratio may be another influential factor of adverse pregnancy outcomes in RIF women with TAI.

## 1. Introduction

Infertility is a common problem, which affects about 10% of the couples of child-bearing age [1,2]. Although assisted reproductive technology is an effective therapy for infertile couples, there are still approximately 10%–15% of the infertile couples failing to conceive after several *in vitro* fertilization and embryo transfer (IVF/ET) cycles. These couples are defined as patients with repeated implantation failure (RIF) [3,4]. Studies have shown that the etiology of RIF commonly includes genetic, hormonal, anatomic, infectious factors and thrombophilic conditions [3]. Recently, accumulating data show that reproductive failure is a condition that can be related with several autoimmune diseases, such as rheumatoid arthritis, systemic lupus erythematosus, and systemic sclerosis [1,5].

Thyroid autoimmunity (TAI), which is defined as the presence of anti-thyroid peroxidase (anti-TPO) and/or anti-thyroglobulin (anti-TG) antibodies, is one of common autoimmunity disorders affecting 5%–20% of normal pregnant women [6,7]. Furthermore, it has been reported that TAI is involved in infertility and pregnancy morbidities [8,9]. The possible mechanisms include (1) TAI induces thyroid dysfunction, which leads to adverse pregnancy outcomes [10], (2) anti-thyroid antibodies directly target human chorionic gonadotropin receptors and some placental antigens to affect implantation [11], (3) TAI serves as not only the necessarily pathogenic antibodies but also as a marker for immune abnormality, which affects reproductive outcomes [12,13,14]. Therefore, the evaluation of immune status in women with TAI becomes very important. It has been reported that TAI is associated with impaired cellular immune responses in women with reproductive failures [12]. However, the association between TAI and immune factors affecting reproductive outcomes in women with RIF remains unclear.

Increased CD3^+^CD4^+^ T helper (Th) cells / CD3^+^CD8^+^ T cytotoxicity (Tc) cells ratio is demonstrated to be related to poor pregnancy outcomes, including recurrent miscarriage (RM) [15], fetal growth restriction (FGR) [16], and preeclampsia [17]. In addition, it is reported that Th/Tc ratio in TAI disease is increased [18]. B cells, as the producer of antibody, play an essential role in pregnancy. Several authors demonstrated that B cells are involved in TAI, and the patients with autoimmune disease have successful pregnancy after B cell depletion therapy [19,20]. Another kind of predominant lymphocyte, natural killer (NK) cells, comprises approximately 15% of blood lymphocytes. Several pieces of evidence show that increased NK cells quantity and NK cells hyperactivity are related to RM or unexplained infertility (UI) [21,22,23]. The NK cells activity is reported to be increased in patients with TAI disease [24]. These studies prompt us to consider that TAI is associated with many abnormal immune factors leading to adverse pregnancy outcomes.

Thus, this study was designed to investigate the prevalence of TAI in women with RIF and its relationship with T cells, B cells and NK cells.

## 2. Materials and Methods

### 2.1. Study Populations

This study was designed as a retrospective cohort study. Women who visited our center from January 2010 to December 2013 were enrolled. All subjects gave their informed consent for inclusion before they participated in the study. The study was conducted in accordance with the Declaration of Helsinki, and the protocol was approved by the Ethics Committee of Shenzhen Zhongshan Urology Hospital. The study group was formed based on the following criteria: inclusion criteria—the patients who had three or more IVF failures with at least one high-quality embryo transferred in per cycle [3]. Exclusion criteria included: (1) the chromosome karyotypes of the couple were abnormal, (2) anatomical tests (ultrasound and hysterosalpingogram /hysteroscopy) were abnormal, (3) infectious disease tests including human immunodeficiency virus (HIV), hepatitis B virus (HBV), hepatitis C virus (HCV), rapid plasma regain (RPR), treponema pallidum particle assay (TPPA), toxoplasma, rubella virus, cytomegalovirus, and herpes virus were positive, (4) basal hormone level including follicle-stimulating hormone (FSH), luteinizing hormone (LH), estradiol (E2), prolactin (PRL), and testosterone (T) were abnormal, and (5) male factor infertility.

At last, 72 women met the criteria. No one was pregnant or on medical treatment when blood was drawn for laboratory evaluation. The presence of anti-thyroid antibodies, the level of thyroid stimulating hormone (TSH) and free thyroxine 4 (FT4), the absolute number and percentage of T, B and NK lymphocytes were assessed in all of the study populations.

In this study, study populations were divided into two groups, RIF women with and without TAI, according to the positivity of anti-thyroid antibodies. There were no significant differences in age, BMI, and basal sex hormone level (FSH, LH, E2, PRL, and T) between the two groups (Table 1).

**Table 1:** Basic clinical characteristics of repeated implantation failure (RIF) women with or without thyroid autoimmunity (TAI).

Characteristics	RIF Women with TAI (*n* = 21)	RIF Women without TAI (*n* = 51)	*p*
Age (years)	33.0 (31.0–37.0)	34.0 (31.0–38.0)	0.490
BMI (kg/m^2^)	21.8 (19.5–23.4)	21.4 (20.0–22.1)	0.848
TSH (uIU/mL)	2.2 (2.0–2.5)	2.2 (1.7–2.5)	0.376
**Reproductive hormone**			
FSH (mIU/L)	6.2 (5.7–7.2)	6.2 (4.8–6.6)	0.299
LH (mIU/L)	4.0 (2.7–4.3)	3.6 (2.6–4.0)	0.587
E2 (pg/mL)	38.0 (29.5–65.7)	47.0 (30.0–64.5)	0.828
PRL (ng/mL)	30.0 (16.3–33.5)	22.1 (15.2–33.5)	0.554
T (ng/mL)	1.1 (0.3–3.0)	0.7 (0.3–3.0)	0.693

RIF: recurrent implantation failure, BMI: body mass index, TSH: thyroid stimulating hormone, FSH: follicle-stimulating hormone, LH: luteinizing hormone, E2: estradiol, PRL: prolactin, T: testosterone.

### 2.2. Thyroid Function Test

The blood of patients was collected during the mid-luteal period. The parameters of thyroid function tests including TSH and FT4 were measured using chemiluminescence assay. TSH level >2.5 uIU/mL was consider to be thyroid function abnormality in women who is planning to have a baby.

### 2.3. Anti-Thyroid Antibodies Assay

Anti-thyroid antibodies including anti-TPO and anti-TG antibodies were determined by a commercial qualitative ELISA kit (INOVA Diagnostics, San Diego, CA, USA).

### 2.4. Peripheral Blood Lymphocytes Assay

The absolute number and percentage of T, B and NK lymphocytes were determined by using the Multitest 6-color TBNK Reagent Kit (BD Biosciences, Franklin Lakes, New Jersey, NJ, USA). In brief, 20 μL of 6-color TBNK reagent including CD3-FITC, CD16/CD56-PE, CD45-PerCP, CD4-PE-Cy^TM^7, CD19-APC and CD8-APC-Cy7 was added into the bottom of the count tube. Then 50 μL of well-mixed, anticoagulated whole blood was pipette into the bottom of the count tube. After incubation with the antibodies for 15 mins, the cells were lysed using 1×FACS lysing solution (BD Biosciences), then acquired and analyzed on the FACSCanto II flow cytometer using the FACSCanto clinical software version 2.2(BD Biosciences).

### 2.5. Statistical Analysis

Statistical analysis was performed with SPSS 17.0 software (SPSS Inc., Chicago, IL, USA). The basic characteristics of study populations were presented as median (quartiles) and difference between median values were determined by non-parametric Mann-Whitney U-test. The difference of the prevalence of thyroid function abnormality between two groups was assessed with chi-square test. The absolute number and percentage of T, B and NK lymphocytes were presented as mean ± S.D. and differences between two groups were determined by independent *t*-test. Differences were considered significant if *p* < 0.05.

## 3. Results

### 3.1. Prevalence of TAI in Women with RIF

Of all of the 72 RIF women investigated, 6.9% (5/72) were TPO^+^TG^−^, 6.9% (5/72) were TPO^−^TG^+^ and 15.3% (11/72) were TPO^+^TG^+^. According to above results, we found 29.2% (21/72) were RIF women with TAI (including TPO^+^TG^-^, TPO^-^TG^+^ and TPO^+^TG^+^), and 70.8% (51/72) were RIF women without TAI (TPO^−^TG^−^) in this study (data not shown in Table).

### 3.2. Association of TAI with Thyroid Function Abnormality in Women with RIF

The prevalence of thyroid function abnormality in RIF women with TAI was higher than that in RIF women without TAI (23.8% *vs.* 13.7%); however, this difference was not significant (*p* > 0.05, Table 2).

**Table 2:** Association of TAI and thyroid function abnormality in women with RIF.

	RIF Women with TAI (*n* = 21)	RIF Women without TAI (*n* = 51)	χ^2^	*p*
Thyroid function abnormality	23.8% (5)	13.7% (7)	0.484	0.487
Thyroid function normality	76.2% (16)	86.3% (44)		

Thyroid function abnormality: TSH level >2.5 uIU/mL.

### 3.3. Association of TAI with Lymphocytes in Women with RIF

**Figure 1:**
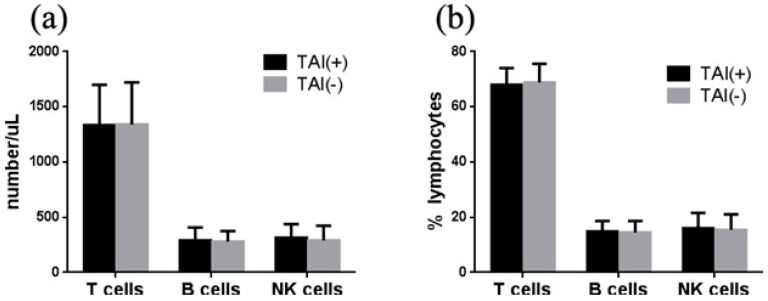
Association of TAI with lymphocytes in women with RIF. (**a**) The absolute number of T, B, and NK cells was not significantly different between RIF women with and without TAI. (**b**) The percentage of T, B, and NK cells was similar between RIF women with and without TAI.

To compare the immune status between RIF women with and without TAI, this study evaluated the levels of lymphocytes in all study populations. The absolute number of CD3^+^ T cells (1334 *vs.* 1340 /μL), CD19^+^ B cells (291 *vs.* 280 /μL) and (CD16+CD56)^+^ NK cells (314 *vs.* 291 /μL) were not significantly different between RIF women with and without TAI, respectively (All *p* > 0.05) (Figure 1a). The percentages of these cells were also similar between these two groups (CD3^+^ T: 68.0 *vs.* 68.9%, CD19^+^ B: 14.8 *vs.* 14.5%, (CD16+CD56)^+^ NK: 16.0 *vs.* 15.4%, all *p* > 0.05) (Figure 1b).

### 3.4. Association of TAI with T Subpopulations in Women with RIF

**Figure 2:**
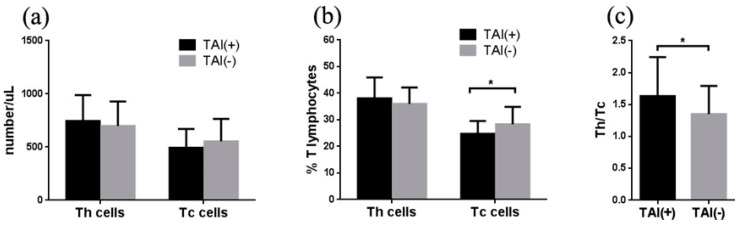
Association of TAI with T subpopulations in women with RIF. (**a**) The number of Th and Tc cells was not significantly different between RIF women with and without TAI. (**b**) The percentage of Tc cells in RIF women with TAI was significantly lower than that in RIF women without TAI, while the percentage of Th cells was not different between these two groups. (**c**) The ratio of Th/Tc was higher in RIF women with TAI, when compared to RIF women without TAI.

To further investigate the association of TAI with T subpopulations, the level of CD3^+^CD4^+^ Th cells and CD3^+^CD8^+^ Tc cells were evaluated in this study. The number of Th cells in RIF women with TAI was higher than those in RIF women without TAI (746 *vs.* 698 /μL), whereas the number of Tc cells was lower (494 *vs* 553 /μL). However, the difference did not reach a statistically significant level (both *p* > 0.05) (Figure 2a). The percentage of Tc cells in RIF women with TAI was significantly lower than that in RIF women without TAI (24.8% *vs.* 28.3%, *p* < 0.05), while the percentage of Th cells was similar between these two groups (38.1% *vs.* 36.0%, *p* > 0.05) (Figure 2b). Furthermore, the ratio of Th/Tc in RIF women with TAI was higher than that in RIF women without TAI (1.63 *vs.* 1.45, *p* < 0.05) (Figure 2c).

## 4. Discussion

The association between TAI and reproductive failure has attracted attention for nearly two decades [25,26]. Some studies reported that TAI affected reproductive outcomes through leading thyroid dysfunction [10], whereas some other studies demonstrated that TAI represented a marker for immune abnormality that was responsible for pregnancy loss [12,13]. However, the thyroid function and immune status of RIF women with TAI remains unclear. In this study, we reviewed thyroid function and lymphocytes in two groups of patients to explore the relationship between thyroid function, lymphocytes and TAI in women with RIF. For the first time, we presented that the percentage of CD3^+^CD8^+^ Tc cells was decreased in RIF women with TAI compared to that in RIF women without TAI. Accompanying this result, an increased Th/Tc ratio was observed in RIF women with TAI.

It has been reported that the prevalence of anti-thyroid antibodies in women with recurrent IVF failure is about 20%–50% in several studies [27,28,29]. Consistent with these results, we found 29.2% of RIF women were positive for anti-thyroid antibodies in this study. The prevalence of thyroid function abnormality in RIF women with TAI was 23.8%, which was not different from that in RIF women without TAI. It indicated that thyroid function abnormality was not the only cause of RIF in women with TAI.

To further investigate the potential cause of RIF in women with TAI, we evaluated some lymphocytes in RIF women with or without TAI. Lower percentage of CD3^+^CD8^+^ Tc and higher Th/Tc ratio has been linked to poor pregnancy outcome, including recurrent spontaneous abortion (RSA) [15,30], fetal growth restriction (FGR) [16], and preeclampsia [17]. In this study, we found that the percentage of CD3^+^CD8^+^ Tc cells was decreased and Th/Tc ratio was increased in RIF women with TAI, when compared to RIF women without TAI. Consistent with these results, an increased Th/Tc ratio has been observed in the TAI disease including Graves disease [31] and postpartum thyroiditis [18], when compared to the healthy control subjects. Th cells mediated humoral or cellular immunity, whereas Tc cells suppressed the activity of Th cells. Stagnaro-Green *et al.* [32] and Xia *et al.* [33] considered that the decreased Tc cells and the higher ratio of Th/Tc may lead to dysfunction of immune monitoring and induce TAI. Similarly, it was reported that decreased regulator T cells were involved in spontaneous miscarriage of patients with rheumatoid arthritis [5]. These results together indicated that the imbalance of T subpopulations may be involved in adverse pregnancy outcomes in patients with autoimmune diseases. Our results indicated that decreased Tc and increased Th/Tc ratio may be another pathogenesis of RIF women with TAI.

Moreover, the Th1/Th2 ratio may shift to a Th1-type response during pregnancy and this change may lead to implantation failure [34]. Ozlem Turhan Iyidir *et al.* [35] demonstrated that during the first trimester, some Th1-associated cytokine levels were significantly higher in women with TAI than those of the women without TAI. In addition, it was reported that depletion of CD3^+^CD8^+^ Tc cells would increase the ratio of Th1/Th2 in mice [36]. These results together indicated that decreased Tc cells might lead to the adverse pregnancy outcome in RIF women with TAI by increasing the ratio of Th1/Th2. Thus, the correlation between the ratio of Th1/Th2 and TAI in RIF women should also attract our attention.

B cells and the antibodies they produced are shown to actively participate in pregnancy [37]. It was reported that B cells alternation was correlated with systemic lupus erythematosus [38]. However, the role of B cells in TAI-induced infertility is still controversial [13]. In our study, CD19^+^ B cells quantity was not significantly different between RIF women with and without TAI. Therefore, B cells may be not the pathogenesis of RIF women with TAI.

Several pieces of evidence linked increased NK cells amount, NK cells hyperactivity and elevated cytotoxic NK cells into RSA or unexplained infertility (UI) [22,39]. It also has been reported that NK cells abnormality was involved in autoimmune thyroid disease [24]. Kim *et al.* [12] demonstrated that the percentage of NK cells was significantly higher in reproductive failure women with TAI, when compare to those of reproductive failure women without TAI. However, our study showed that the quantity of NK cells was not significantly different between RIF women with and without TAI. The discrepancies in these studies may be partly because of different study populations. The study population in the report of Kim *et al.* was women with RSA or UI [12], whereas our study population was women with RIF.

In summary, this study observed that thyroid function abnormality was not correlated with TAI in RIF women; however, Tc cells quantity was decreased and Th/Tc ratio was increased in RIF women with TAI, when compared to those inRIF women without TAI.

## 5. Conclusions

This study indicated that TAI may be a marker for the T subpopulations’ abnormality that leads to RIF. These data provided a new perspective for understanding the pathogenesis of RIF women with TAI. In addition, considering the previous studies revealing the therapeutic potential of immunosuppressors such as tumor necrosis factor-alpha inhibitor in autoimmune diseases with immunology abnormality, further studies should be aimed at improving knowledge about the immunotherapy strategies in RIF women with TAI.
